# Does Exercise Performance Differ Among Male Law Enforcement Officers Based on Their Body Mass Index Category?

**DOI:** 10.3390/healthcare13131584

**Published:** 2025-07-02

**Authors:** Drew E. Gonzalez, Don R. Melrose, Filip Kukić, Robert G. Lockie, Robin M. Orr, J. Jay Dawes

**Affiliations:** 1 Department of Kinesiology and Sport Management, Texas A&M University, College Station, TX 77843, USA; dg18@tamu.edu; 2 Department of Kinesiology, Texas A&M University-Corpus Christi, Corpus Christi, TX 78412, USA; don.melrose@tamucc.edu; 3 Faculty of Physical Education and Sports, University of Banja Luka, 78000 Banja Luka, RS, Bosnia and Herzegovina; filip.kukic@ffvs.unibl.org; 4 Department of Kinesiology, California State University Fullerton, Fullerton, CA, 92831, USA; rlockie@fullerton.edu; 5 Tactical Research Unit, Bond University, Robina, QLD 4226, Australia; rorr@bond.edu.au; 6 School of Kinesiology, Applied Health, and Recreation, Oklahoma State University, Stillwater, OK 74078, USA; 7 Tactical Fitness and Nutrition Lab, Oklahoma State University, Stillwater, OK 74078, USA

**Keywords:** tactical athletes, tactical strength and conditioning, police officers, cardiometabolic health

## Abstract

Objectives: To assess differences in exercise performance among law enforcement officers (LEOs) based on body mass index (BMI). Methods: Five hundred and thirty-two male LEOs (*n* = 532; age 38.9 ± 7.4 yrs; height: 180.1 ± 6.9 cm; body mass: 92.1 ± 15.1 kg) were analyzed. The LEOs were stratified into three BMI groups: “healthy” (18.5–24.9 kg/m^2^), “overweight” (25.0–29.9 kg/m^2^), and “obese” (≥30.0 kg/m^2^). Tests for push-ups, sit-ups, estimated VO_2_max, vertical jump (VJ), and peak anaerobic power output (PAPw) were conducted. Non-parametric Kruskal–Wallis H and Quade’s rank-based ANCOVA with age as a covariate test were used to evaluate differences in exercise performance between BMI groups. Mann–Whitney U tests with Bonferroni post hoc corrections were used for pairwise comparisons. Effect sizes were calculated as rank eta squared (ηH^2^) for the Kruskal–Wallis H test results. Results: Differences were noted across BMI groups for the push-ups (*p* < 0.001, ηH^2^ = 0.101), sit-ups (*p* < 0.001, ηH^2^ = 0.187), VO_2_max (*p* < 0.001, ηH^2^ = 0.145), VJ (*p* < 0.001, ηH^2^ = 0.137), and PAPw (*p* < 0.001, ηH^2^ = 0.504). The pairwise comparisons revealed differences between each group, with the obese and overweight groups exhibiting a lower VJ, VO_2_max, and performance in push-ups and sit-ups while having a higher PAPw than the healthy group, even after adjusting for age. Conclusions: These data demonstrate that a higher BMI is associated with poorer exercise performance, except for PAPw, and highlight the importance of maintaining a healthy BMI in LEOs.

## 1. Introduction

Research among law enforcement officers (LEOs) has consistently reported that body mass tends to increase with years of service [1,2,3]. Studies have also demonstrated that most law enforcement duties are approximately 80–90% sedentary, due to long hours spent sitting in the patrol unit and completing paperwork [4,5], which can further intensify occupational demands relative to one’s physical preparedness and alertness, as intervals of sitting or inactivity typically precede strenuous activities. The sudden change in habitual conditions may increase the risk of injuries, morbidity, and even premature mortality, especially for LEOs in poor physical health [6].

Many law enforcement agencies conduct routine physical and fitness assessments, during which anthropometric measurements, such as weight, height, and body composition parameters, are recorded [1,6,7,8,9,10,11,12,13,14]. Information regarding the individual’s body mass index (BMI), waist-to-hip, and waist-to-height ratio may be calculated from these values [10,15]. Body mass index is a quantitative measure that estimates one’s body mass in relation to one’s height [16,17]. Although there are benefits to using BMI as a general screening tool for one’s health (i.e., quick and cost-effective), the BMI scale has received criticism for its inability to differentiate between fat mass (FM) and skeletal muscle mass (SMM) [10,18,19]. Noting this limitation in tissue differentiation, for tactical personnel, a higher BMI has been shown to unfavorably affect performance in running, push-ups, sit-ups, and vertical jump (VJ) [20]. Dawes et al. [12] demonstrated that LEO trainees in a ‘healthy’ BMI group had significantly higher Defensive Tactics and Arrest Control scores than those in an ‘overweight’ BMI group. Similarly, negative correlations have been found between the BMI category and 1.5 km run performance and maximal oxygen uptake (VO_2_max) [11,21]. When longer distances were analyzed (e.g., 3 km), BMIs over 30 kg/m^2^ demonstrated a negative association with running velocity [4]. Furthermore, Dawes et al. [11] found that trunk muscular endurance was negatively correlated with BMI in part-time special weapons and tactics (SWAT) officers. Based on previous studies, a higher BMI, regardless of body composition [20], negatively influences certain physical fitness measures. Hence, the BMI within tactical populations may be an accurate, quick, and non-invasive method to provide an initial health and fitness evaluation.

Law enforcement agencies in the US are currently not held to a national standard of physical fitness. However, The Cooper Institute proposed diverse assessments for this population [22]. While these assessments do not provide a comprehensive scope of a LEO’s ability to perform the occupation, they still offer pertinent information regarding the individual’s risk of injury and ability to perform specific occupational duties. For example, research suggests that poor scores in VJ height and aerobic fitness tests (i.e., 2.4 km run and 20 m multistage fitness test [20MFST]) are strongly correlated to musculoskeletal injury risk [23,24]. For instance, Orr and colleagues [23] assessed the relationship between leg power (measured via VJ height) and rates of reported injuries among 1201 basic police recruits in an Australian Police Force College. The research team found that 17% of participants who reached a 60–70 cm VJ height reported injuries or illnesses, while 54% of those who reached a 30–34 cm VJ height reported injuries and illnesses, highlighting a threefold greater risk of injury or illness for those with a lower VJ height. Furthermore, in a sample of 307 police trainees [25], push-ups (r_s_ = −0.621), VJ (r_s_ = −0.515), and 2.4 km run time (r_s_ = −0.698) were all correlated with performance on an occupationally specific physical capability test that involved pushing and dragging objects, vaulting walls, and sprinting [26] Concerning BMI, Dawes et al. [13] investigated specific fitness variables that predicted a LEO’s success in a physical ability test (PAT) and observed a significantly higher BMI in the low-performers group than the high-performers, signifying the correlation between BMI and physical performance [13].

Given these findings, BMI can provide important insights into physical and exercise performance-related outcomes among tactical personnel. However, it remains less clear how LEOs across different BMI classifications differ in exercise performance parameters. Therefore, this study aimed to assess differences in exercise performance parameters among LEOs based on BMI classifications. It was hypothesized that those with a higher BMI would have lower fitness levels.

## 2. Materials and Methods

### 2.1. Subjects

Retrospective data of 532 male LEOs (age: 38.9 ± 7.4 years; height: 180.1 ± 6.9 cm; body mass: 92.1 ± 15.1 kg) from a single US-based law enforcement agency were analyzed. The data were derived from a portion of the agency’s fitness assessment conducted in 2018. Demographic data included the LEOs’ age, height, body mass, and BMI. Both height and body mass were self-reported, following a previously conducted method by Dawes et al. [10], showing no difference between self-reported and measured height (*p* = 0.830), body mass (*p* = 0.527), and BMI (*p* = 0.623). The LEOs were stratified into three different BMI classifications, including healthy (18.5–24.9 kg/m^2^), overweight (25.0–29.9 kg/m^2^), and obese (≥30.0 kg/m^2^). This study’s sample size was a convenience sample of de-identified data provided by the LEO agency, and the researchers had no control over the final sample size. The inclusion criteria for the participants included participating in the physical performance testing; however, not all participants were required to complete every test. Therefore, there are differences in the sample size per component of the physical performance variables. The only exclusion criterion for this study was if the LEOs did not complete any of the physical performance testing, which meant they did not have any data reported. Nevertheless, all procedures were conducted according to the Declaration of Helsinki. The Oklahoma State University (#ED-19-120-STW) institutional ethics committee approved using archival data for this study.

### 2.2. Parameters

Specific performance assessments were collected for each group during the agency’s annual fitness assessment, which included 1 min push-ups and sit-ups tests, a 20MFST, and a VJ. The results from 20MFST and VJ were then used in specific formulas, detailed hereafter, to determine VO_2_max and anaerobic power output, respectively. The scores for each test were collected by the law enforcement agency’s state patrol training staff. All staff were trained by an instructor who was a certified Tactical Strength and Conditioning Facilitator (TSAC-F), and TSAC-F-certified instructors verified the proficiency of each staff.

### 2.3. Upper Body and Trunk Muscular Endurance

#### 2.3.1. One-Minute Push-Ups and Sit-Ups

The objective of these assessments was to complete as many push-ups or sit-ups as possible within a 1 min time frame. The validity of these procedures was acknowledged in previous studies [7,27,28]. The test was concluded once the officer failed to maintain proper technique and criteria, or 1 min had expired. Repetitions were counted in single complete units.

#### 2.3.2. Twenty-Meter Multistage Fitness Test (20MSFT)

The protocols for the 20MSFT have been previously described by Dawes et al. [13] and are a commonly used assessment by LEOs/agencies to assess aerobic fitness [26]. The objective of this test was to run between two lines marked on the ground, which were spaced 20 m apart, while keeping pace to an audio cadence. The test started with an initial speed of 8.5 km/h, increasing by 0.5 km/h at every stage. The test was concluded when an officer reached volitional fatigue or could not maintain the stage’s set pace for two consecutive beeps. The total number of completed shuttles was recorded as their final score [29]. The total number of completed shuttles was converted to an estimated VO_2_max to assess aerobic fitness level [30].

### 2.4. Lower Body Power and Peak Power Output

#### Vertical Jump (VJ) Height

Similar to previous investigations in this population, VJ height was measured using a Just Jump (ProBotics Inc., Huntsville, AL, USA) system that calculates jump height based on flight time [7,27,28]. Each officer stepped onto the mat and, when ready, performed a vertical jump with an arm swing. Three attempts were allowed, with 10–30 s of rest between jumps. The highest jump was recorded as the officer’s final score and was then converted to centimeters for analysis. Additionally, the best VJ attempt was used to calculate peak anaerobic power output (PAPw) using the Sayers equation: Peak Power (W) = (60.7 × jump height (cm)) + (45.3 × body mass (kg)) − 2055 [31]. Relative PAPw was also calculated: $\frac{PAPw}{kg}$. It is worth noting that VJ testing is fairly common among LEOs and is a practical and cost-effective method for assessing PAPw [32].

### 2.5. Statistical Analysis

#### 2.5.1. Overview

All statistical analyses were performed with SPSS statistical analysis software (IBM Corp., Armonk, NY, USA, Version 29). Statistical significance was accepted at *p* ≤ 0.05 for all statistical analyses. Data are reported as medians and interquartile ranges for all nonparametric test results, as well as means ± standard deviations.

#### 2.5.2. G*Power Sensitivity Analysis

Given that we used a large convenience sample (n = 532), conducting an a priori power analysis was not feasible. Rather, a post hoc sensitivity analysis was performed using G*Power (v3.1.9.6) to determine the minimum detectable effect sizes across the primary parameters. For non-parametric group comparisons (e.g., Kruskal–Wallis), the parametric analog (one-way ANOVA) indicated that, assuming α = 0.05 and 80% power, the study was sensitive to detect effect sizes of f = 0.135, reflecting small-to-moderate group differences. Similarly, for Quade’s rank-based ANCOVA, sensitivity analysis using fixed-effects ANCOVA (with 3 groups and 1 covariate) yielded a minimum detectable effect size of f = 0.135. For correlational analyses, sensitivity analysis based on Pearson’s r (used as an approximation for Spearman’s rank-order correlation test) indicated that the study could detect correlation coefficients of r ≥ ±0.085, indicating sufficient power to detect small to moderate associations. These analyses provide conservative estimates of statistical sensitivity and are reported here to increase transparency regarding our sample adequacy.

#### 2.5.3. Normality and Correlation Analysis

Given our study sample size (exceeding 50 participants), the Kolmogorov–Smirnov Test was used to assess normality, which was violated for all demographic and exercise performance parameters except power output. A Spearman’s rank-order correlation test was used to evaluate the association between the demographic and exercise performance variables, with a Pearson’s correlation test conducted on PAPw.

#### 2.5.4. Non-Parametric Tests

A non-parametric Kruskal–Wallis H test was used (as normality was violated) to assess differences in exercise performance parameters between BMI classification groups. Where statistically significant effects were found, the Mann–Whitney U test was used for pairwise comparisons. To reduce the risk of Type 1 errors (false positives), Bonferroni post hoc corrections for the Mann–Whitney U test were interpreted. Effect sizes were calculated as rank eta squared (ηH^2^), which was calculated using the following formula, where H is the Kruskal–Wallis H test statistic and n is the total number of observations: ηH^2^ = $\frac{H}{\left(n-1\right)}$. For pairwise comparisons between groups (i.e., healthy versus obese), effect sizes were calculated using the rank-biserial correlation (r_b_), where the following formula was applied, with N representing the total sample size of the two groups being compared: r_b_ = $\frac{z}{\sqrt{N}}$. The r_b_ was interpreted as follows: 0.1 = small, 0.3 = medium, 0.5 = large. In addition, a univariate non-parametric Quade’s rank-based ANCOVA test (an alternative to a parametric ANCOVA as normality was violated) was conducted with age as a covariate, and pairwise comparisons were performed using *t*-tests on ranked covariate-adjusted residuals. The effect size for the ANCOVA test results was computed via the following equation based on the ranked data: r_ANCOVA_ = tt^2+df. The data r_ANCOVA_ is interpreted the same as the r_b_.

#### 2.5.5. Parametric Tests

A one-way analysis of variance (ANOVA) was conducted to assess the difference in PAPw between BMI classification groups, given that PAPw did not violate the assumption of normality. The effect size for ANOVA statistics was calculated as partial eta squared. For pairwise comparisons with Bonferroni post hoc corrections between groups, effect sizes were calculated using Cohen’s d and interpreted as follows: <0.2 trivial, <0.5 small, <0.8 moderate, <1.2 large, and >1.2 very large [33]. Then, to account for age as a covariate, an analysis of covariance (ANCOVA) was conducted, with pairwise comparisons using the Bonferroni post hoc correction.

#### 2.5.6. Multicollinearity

To assess potential multicollinearity between BMI and age, variance inflation factors (VIFs) were calculated using linear regression models. The VIFs were interpreted as <2 = low multicollinearity, 2 to 5 = moderate multicollinearity, and >5 = high multicollinearity.

## 3. Results

### 3.1. Correlation Analysis

Spearman’s rank-order and Pearson’s (PAPw only) correlation analysis revealed significant (*p* < 0.001) positive and inverse associations between age, height, body mass, BMI, and exercise performance parameters (See Table 1). Notably, correlations were strongest between body mass and PAPw (r = 0.956), followed by BMI and PAPw (r = 0.807) and relative PAPw and VJ (r_s_ = 0.710). Weak to moderate inverse correlations were noted between BMI and VJ height (r_s_ = −0.396), sit-ups (r_s_ = −0.446), push-ups (r_s_ = −0.328), and VO_2_max (r_s_ = −0.541).

### 3.2. Demographic Data

Significant differences were noted across BMI classification groups for the demographic variables, including age (*p* < 0.001, ηH^2^ = 0.050), body mass (*p* < 0.001, ηH^2^ = 0.607), and BMI (*p* < 0.001, ηH^2^ = 0.843), while height did not differ (*p* = 0.529, ηH^2^ = 0.002). Pairwise comparisons revealed that the healthy BMI group differed from the obese group in that they were younger (*p* < 0.001, r_b_ = −0.312), as well as having a lower body mass (*p* < 0.001, r_b_ = −1.051) and a lower BMI (*p* < 0.001, r_b_ = −1.247). In addition, the healthy BMI group differed from the overweight group in that they were younger (*p* = 0.006, r_b_ = −0.163), as well as having a lower body mass (*p* < 0.001, r_b_ = −0.428) and BMI (*p* = 0.006, r_b_ = −0.163). Lastly, the overweight BMI group differed from the obese group in that they were younger (*p* = 0.006, r_b_ = −0.138), as well as having a lower body mass (*p* < 0.001, r_b_ = −0.596) and BMI (*p* < 0.001, r_b_ = −0.683).

The rank-based ANCOVA analysis revealed significant differences across the BMI groups for body mass (*p* < 0.001) and BMI (*p* < 0.001), but not height (*p* = 0.534), when accounting for age as a covariate. Pairwise comparisons revealed that the healthy BMI group had a lower body mass (*p* < 0.001, r_ANCOVA_ = −0.737) and BMI (*p* < 0.001, r_ANCOVA_ = −0.885) compared to the obese group. Furthermore, the healthy BMI group had a lower body mass (*p* < 0.001, r_ANCOVA_ = −0.449) and BMI (*p* < 0.001, r_ANCOVA_ = −0.677) compared to the overweight group. The overweight BMI group also had a lower body mass (*p* < 0.001, r_ANCOVA_ = −0.619) and BMI (*p* < 0.001, r_ANCOVA_ = −0.799) compared to the obese group. Table 2 displays demographic data.

### 3.3. Exercise Performance Measures

**Figure 1:**
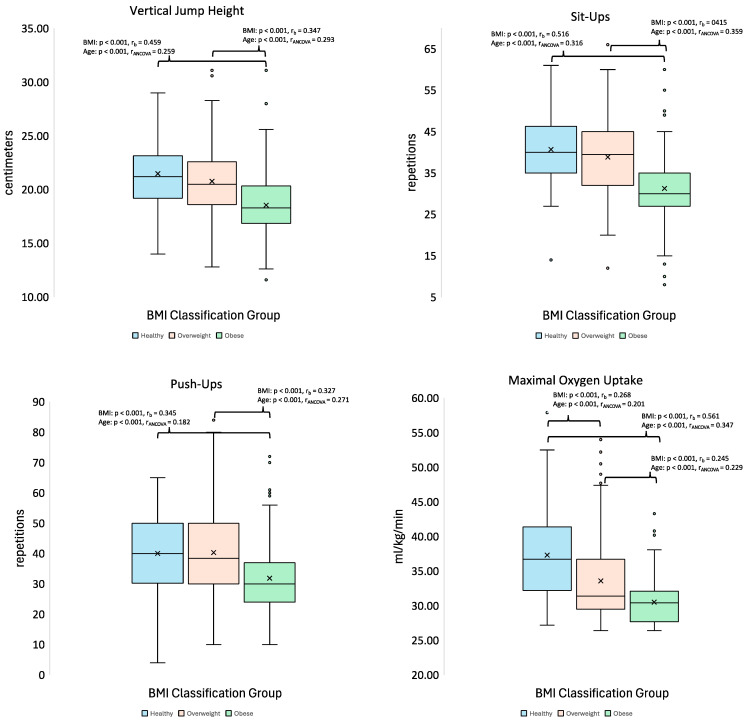
Exercise performance data (except power output); ‘age’ denotes the effect of BMI when adjusting for age as a covariate. r_b_ = rank-biserial correlation effect size and r_ANCOVA_ = the ANCOVA test ranked data effect size.

**Figure 2:**
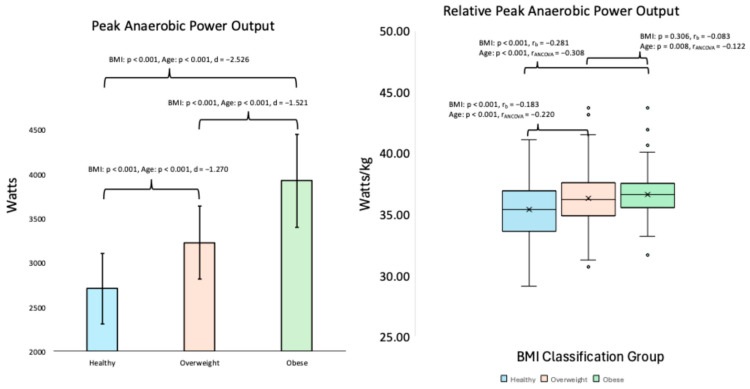
Absolute (parametric; mean ± SD) relative (non-parametric; Median and Interquartile ranges) peak anaerobic power output data; ‘age’ denotes the effect of BMI when adjusting for age as a covariate. r_b_ = rank-biserial correlation effect size and r_ANCOVA_ = the ANCOVA test ranked data effect size.

The non-parametric and parametric ANOVA analyses revealed significant differences were noted across BMI classification groups for push-ups (*p* < 0.001, ηH^2^ = 0.101), sit-ups (*p* < 0.001, ηH^2^ = 0.187), VO_2_max (*p* < 0.001, ηH^2^ = 0.145), VJ height (*p* < 0.001, ηH^2^ = 0.137), absolute PAPw (*p* < 0.001, η_p_^2^ = 0.485), and relative PAPw (*p* < 0.001, ηH^2^ = 0.040). Pairwise comparisons revealed the healthy BMI group differed from the obese group in that they completed more push-ups (*p* < 0.001, r_b_ = 0.345) and sit-ups (*p* < 0.001, r_b_ = 0.516), had higher VO_2_max values (*p* < 0.001, r_b_ = 0.561) and VJ height (*p* < 0.001, r_b_ = 0.459), but had lower absolute PAPw (*p* < 0.001, d = −2.526). In addition, the healthy BMI group differed from the overweight group in that they had higher VO_2_max values (*p* < 0.001, r_b_ = 0.268) and had lower absolute PAPw (*p* < 0.001, d = −1.270). The overweight BMI group differed from the obese group in that they completed more repetitions for the push-ups test (*p* < 0.001, r_b_ = 0.327) and sit-ups test (*p* < 0.001, r_b_ = 0.415), in addition having displayed higher VO_2_max values (*p* < 0.001, r_b_ = 0.245) VJ height (*p* < 0.001, r_b_ = 0.347), but lower absolute PAPw (*p* < 0.001, d = −1.521). Lastly, the healthy BMI group had lower relative PAPw than the overweight BMI group (*p* < 0.001, r_b_ = −0.183) and obese BMI group (*p* = 0.003, r_b_ = −0.281), with no difference noted between the overweight and obese BMI groups (*p* = 0.306, r_b_ = −0.083). Table 3 presents the exercise performance parameters as raw data, means, and standard deviations. Figure 1 displays the differences between BMI groups in exercise performance data between BMI categories using the nonparametric ANOVA and ANCOVA test results. Figure 2 displays the differences between BMI groups in both absolute (parametric) and relative PAPw (non-parametric) data, categorized by BMI, along with the ANOVA and ANCOVA test results.

The rank-based ANCOVA analysis revealed significant differences across the BMI groups for push-ups (*p* < 0.001), sit-ups (*p* < 0.001), VO_2_max (*p* < 0.001), VJ height (*p* < 0.001), and the parametric ANOCOVA test revealed differences for PAPw (*p* < 0.001) when accounting for age as a covariate. Pairwise comparisons revealed that the healthy BMI group completed more repetitions for the push-ups test (*p* < 0.001, r_ANCOVA_ = 0.182) and sit-ups test (*p* < 0.001, r_ANCOVA_ = 0.316), as well as had higher VO_2_max values (*p* < 0.001, r_ANCOVA_ = 0.347) and VJ height values (*p* < 0.001, r_ANCOVA_ = 0.259) compared to the obese group. Furthermore, the healthy BMI group had higher VO_2_max values (*p* < 0.001, r_ANCOVA_ = 0.201) than the overweight BMI group. The overweight BMI group also completed more repetitions for the push-ups test (*p* < 0.001, r_ANCOVA_ = 0.271) and sit-ups test (*p* < 0.001, r_ANCOVA_ = 0.359), as well as had higher VO_2_max values (*p* < 0.001, r_ANCOVA_ = 0.229) and VJ height values (*p* < 0.001, r_ANCOVA_ = 0.293) compared to the obese group. Lastly, the obese BMI group exhibited higher relative PAPw than both the healthy BMI group (*p* < 0.001, r_ANCOVA_ = −0.308) and the overweight BMI group (*p* = 0.008, r_ANCOVA_ = −0.122), with the overweight BMI group also displaying a higher relative PAPw than the healthy BMI group (*p* < 0.001, r_ANCOVA_ = −0.220). In terms of absolute PAPW, the parametric ANCOVA analysis revealed that the obese BMI group had a higher absolute PAPw than the healthy BMI group (*p* < 0.001) and the overweight BMI group (*p* < 0.001), in addition to the overweight BMI group displayed a higher absolute PAPw than the healthy BMI group (*p* < 0.001).

### 3.4. Multicollinearity Assessment

The multicollinearity assessment revealed consistent VIFs for BMI and age across models (VIF ≈ 1.025 to 1.060), which suggests no issue of multicollinearity. These findings support the interpretation that BMI and age contributed independently to exercise performance outcomes.

## 4. Discussion

This retrospective study examined differences in exercise performance among LEOs according to BMI classifications. The main findings of the present study demonstrated that a higher BMI was associated with poorer exercise performance, except for estimated lower body peak power output, even when accounting for age as a covariate. Thus, the hypothesis of this study was generally supported. The obese and overweight BMI groups displayed lower push-up scores (22.6% and 0.6%), sit-up scores (26.1% and 4.6%), VO_2_max values (20.0% and 10.5%), and VJ height (14.5% and 3.2%), while having a greater absolute PAPw (36.7% and 17.5%) and relative PAPw (3.3% and 2.5%) than the healthy group, respectively. It is also important to note that the overweight and obese BMI groups were older than the healthy BMI group (7.3% and 12.7% older, respectively). Spearman’s rank-order correlation results showed that BMI was weakly to moderately inversely correlated to the exercise performance parameters, excluding PAPw, which was strongly positively correlated with BMI. On the contrary, age was only weakly negatively correlated with push-ups and moderately positively correlated with PAPw. It is worthwhile noting that the correlation between BMI and absolute PAPw indicates that individuals with greater body mass tend to produce higher absolute power outputs. This relationship is consistent with fundamental biomechanical principles, as increased mass can contribute to greater force production during high-intensity physical tasks. Collectively, our findings suggest that the LEO with a higher BMI consistently performed worse across all exercise performance parameters. This information may be useful in developing nutritional and exercise intervention strategies to improve one’s BMI and, thus, improve exercise performance outcomes.

While differences were noted between the healthy BMI group and the two other BMI groups, the percentage differences and larger effect sizes for the exercise performance parameters demonstrate that, on average, obese LEOs performed worse on their fitness tests. However, PAPw was an exception, as revealed by a strong positive correlation between absolute PAPw and BMI. An underlying explanation for this is that a greater BMI—particularly when accompanied by increased fat-free mass—can enhance absolute power output, as more muscle mass contributes to greater force production during short-duration, high-intensity tasks. However, this is only speculative concerning our sample, as fat-free mass was not directly assessed. Furthermore, this may also underscore the importance of evaluating PAPw relative to body mass (e.g., watts/kg), particularly in occupational settings where relative power may be a more relevant indicator of functional capacity and task performance. Yet, we found that the healthy BMI group also displayed a lower relative PAPw than the other BMI groups, which may be explained by the notion that the LEOs with a higher BMI may still possess a higher level of skeletal muscle mass than their lower BMI counterparts. Nevertheless, our findings suggest that being classified as obese (BMI ≥ 30 kg/m^2^) is associated with a meaningful reduction in exercise performance and, potentially, occupation-specific physical abilities [13]. This is consistent with previous reports demonstrating that LEOs with a higher BMI have lower exercise performance parameters than their healthy BMI counterparts. For instance, Dawes and colleagues [12] assessed the influence of BMI classification on the physiological and perceptual demands of defensive tactics training among 24 state patrol officers, finding that overweight officers had lower defensive tactics scores than their counterparts with a healthy BMI. Interestingly, Estevez et al. [34] found that mildly obese Brazilian male highway police officers (28.6 ± 4.8 kg/m^2^) did not experience a negative impact on their aerobic power or muscular endurance performance based on their BMI classification. The discrepancy between our findings and those of Dawes and Estevez could be attributed to numerous factors, including differences in sample size, BMI range, and classification. For instance, the Estevez study classified participants as mildly obese, and the large standard deviation (±4.8 kg/m^2^) suggests substantial variability, which may have masked potential BMI-related performance effects. In our study, the larger sample size and classification of individuals with higher BMI levels (≥30 kg/m^2^) likely allowed for clearer detection of performance decrements associated with obesity. Nonetheless, it is plausible that LEOs with higher BMI may encounter increased physiological strain [10,18], which may negatively impact exercise and job performance.

Previous reports have shown that one’s ability to perform a greater number of push-ups [35] and those with a greater cardiorespiratory fitness (CRF) level have a lower incidence of cardiovascular disease (CVD)-related events [36,37,38,39]. An important observation of the present study is that the healthy and overweight BMI group, on average, demonstrated the ability to perform 40 push-ups, which has been identified as a threshold associated with a statistically significantly lower risk of a CVD event occurring among male firefighters (who are a similar occupational group to LEOs) [35]. It is worth noting that not every LEO within these respective BMI groups could complete 40 push-ups (i.e., 56% of the healthy individuals and 49.6% of the overweight individuals completed ≥40 push-ups). Regarding CRF, the LEOs of the present study collectively displayed sub-optimal VO_2_max values (i.e., 29.5 ± 12.3 mL/kg/min), with even the healthy BMI group exhibiting low levels (36.2 ± 9.2 mL/kg/min). Compared to the American College of Sports Medicine VO_2_max value standard (based on age), the healthy BMI group is considered to have “poor” CRF, while the overweight and obese BMI groups have “very poor” CRF levels [40]. It is essential to note that although the method used to estimate VO_2_max has been previously employed [26], and some researchers have considered this method as valid [41,42], it is plausible that VO_2_max values are underestimated [26]. Utilizing indirect calorimetry during a cardiopulmonary exercise test can provide a more accurate assessment of LEOs’ CRF, and it is possible that the healthier BMI group may display higher CRF levels, while the overweight and obese BMI groups may display even poorer CRF. Nonetheless, the results in the current investigation suggest that the LEOs in this study should aim to improve CRF regardless of BMI category.

Several studies have shown that significant increases in body mass, as well as BMI, tend to occur within tactical populations as years of service increase [1,2,3]. This may be due to a variety of factors, such as decreased physical activity and sleep quality, increased levels of stress, irregular work schedules, and reliance on fast food due to erratic shiftwork [2,22]. Additionally, certain aspects of physical performance, such as lower-body power, upper-body strength and endurance, and aerobic fitness, have been shown to decline with age [6]. Therefore, the two-fold effect of an increase in BMI and age may negatively impact an officer’s ability to perform essential job tasks across the occupational lifespan. Noting this, a well-structured conditioning program could help mitigate the loss of fitness with aging and optimize officer BMI. Nevertheless, it is important to note that although the overweight and obese LEOs of the present study were older than those with a healthy BMI, our assessment of age as a covariate in both parametric and non-parametric ANCOVA analyses confirmed that BMI remained a unique, statistically significant, and independent predictor of exercise performance outcomes. Lastly, the multicollinearity analysis revealed no issues of multicollinearity between BMI and age, suggesting that BMI and age contribute independently to performance.

The present study is not without limitations. First, the data come from a large convenience sample and are non-probabilistic; therefore, strict interpretations may not be appropriate. Second, the sample was limited to only males and did not account for other potentially influential factors, such as years of experience, race, and ethnicity. It is plausible that factors such as sex, exercise training history, and years of experience may influence the outcomes of this study. For instance, although speculative, those who log more training hours per week are likely to exhibit greater exercise performance parameter metrics. Additionally, female LEOs may exhibit lower performance values across all BMI groups compared to their male counterparts. Future work should include female LEOs and consider the impact of other population-based variables. It is also worth noting that because this study utilized a convenience sample, we were unable to control for the inter-rater reliability of the testers from the EO agency. Lastly, although Dawes et al. [10] have demonstrated no differences between self-reported and measured height, body mass, and BMI, it is still worth noting this as a potential limitation, as one may still over- or underestimate their metrics.

## 5. Conclusions

While it is well understood that a higher BMI can negatively impact one’s physical ability [43,44], the results of this study highlight the importance of maintaining healthy BMI levels in LEOs, as they may predict the underlying fitness variables necessary for occupational duties. Tactical athletes should focus on employing interventions that aid their ability to maintain a healthy BMI, and further consideration is likely needed to understand the impact of body composition (i.e., fat and lean mass) on exercise performance among LEOs, as BMI does not account for these variables. In terms of practical applications, interventions, such as routine physical evaluations, proper strength and conditioning programs, behavioral strategies for maintaining healthy habits, stress management, sleep quality, and nutritional approaches, should be prioritized in law enforcement agencies. Intervention priority should be given to those with a higher BMI and/or older age, as they are at most risk of poorer task performance on the job.

## Figures and Tables

**Table 1:** Correlation Analysis Results.

Variable	Age	Height	Body Mass	BMI	VJ	Sit−Ups	Push−Ups	VO_2_max	PAPw
Height (cm)	−0.019	−	−	−	−	−	−	−	−
Body mass (kg)	0.197 **	0.504 **	−	−	−	−	−	−	−
Body mass Index (kg/m^2^)	0.249 **	0.017	0.861 **	−	−	−	−	−	−
Vertical Jump (cm)	−0.442 **	−0.052	−0.352 **	−0.396 **	−	−	−	−	−
Sit−Ups (repetitions)	−0.376 **	−0.010	−0.372 **	−0.446 **	0.466 **	−	−	−	−
Push−Ups (repetitions)	−0.344 **	−0.226 **	−0.383 **	−0.328 **	0.501 **	0.663 **	−	−	−
VO_2_max (mL/kg/min)	−0.418 **	0.037	−0.427 **	−0.541 **	0.409 **	0.580 **	0.517 **	−	−
Peak Power Output (watts) ^#^	0.069	0.511 **	0.956 **	0.807 **	−0.063	−0.302 **	−0.269 **	−0.200 **	−
Relative Peak Power Output (watts/kg)	−0.317 **	0.282 **	0.311 **	0.209 **	0.710 **	0.199 **	0.197 **	0.120 *	0.590 **

* Significant at *p* < 0.05; ** Significant at *p* < 0.001. BMI = Body Mass Index; cm = centimeters; kg = kilograms; m = meters; min = minutes; mL = mililiters; VJ = Vertical Jump, VO2max = Maximal Oxygen Uptake. PAPw = Peak Anerobic Power Output. # denotes that the Pearson’s correlation test was used for the peak power output.

**Table 2:** Demographic data.

BMI Classification Group			Age (years)				Height (cm)				Body Mass (kg)				Body Mass Index (kg/m^2^)		
		*N*				*N*				*N*				*N*			
Median (IQR)	Healthy	102	36 (30−41.5) *^†^			102	180.3 (175.2−183.5)			102	74.8 (70.9−80.7) *^†¥§^			102	23.7 (22.8−24.4) *^†¥§^		
	Overweight	259	39 (33−45) *^‡^			260	180.3 (175.2−185.4)			260	88.4 (83.4−92.9) *^‡∞¥^			260	27.3 (26.2−28.6) *^‡∞¥^		
	Obese	170	43 (36−47) ^‡†^			170	180.3 (175.2−183.5)			170	104.3 (99.2−113.3) ^‡†§∞^			170	32.1 (31−33.9) ^‡†§∞^		
	Total	531	39 (33−46)			532	180.3 (175.2−185.4)			532	90.7 (81.6−101.1)			532	28 (25.4−30.9)		
Mean ± SD	Healthy	102	35.93	±	7.62	102	180.7	±	7.18	102	76.1	±	7.72	102	23.27	±	1.39
	Overweight	259	38.88	±	7.07	260	179.66	±	6.72	260	88.43	±	8.49	260	27.35	±	1.46
	Obese	170	40.83	±	7.15	170	180.33	±	7.31	170	107.46	±	12.7	170	32.99	±	2.95
	Total	531	38.94	±	7.39	532	180.07	±	6.99	532	92.15	±	15.14	532	28.37	±	4.06

Data a represented as medians (IQR) for non−parametric test, as well as means ± SD. n sizes vary due to metrics not being completed or missing observations for the participant(s). BMI = Body mass Index (kg/m^2^); cm = circumference; IQR = interquartile range; kg = kilogram; m = meters. The pairwise comparison non−parametric Mann−Whitney U test results are denoted with the following symbols: * Significantly different from Obese group; deviations; ^†^ Significantly different from Overweight group; ^‡^ Significantly different from Healthy group. The univariate non−parametric Quade’s rank−based ANCOVA test results are denoted with the following symbols: ^¥^ Significantly different from Obese group; deviations; ^§^ Significantly different from Overweight group; ^∞^ Significantly different from Healthy group.

**Table 3:** Exercise performance data.

BMI Classification Group		Sit−Ups (Repetitions)				Push−Ups (Repetitions)				VO_2Max_ (mL/kg/min)				Vertical Jump (cm)				Absolute PAPw (watts)				Relative PAPw (watts/kg)		
	*N*				*N*				*N*				*N*				*N*				*N*			
healthy	102	40.69	±	8.14	100	40.05	±	11.79	99	37.32	±	6.85	90	21.47	±	2.95	90	2706.55	±	399.81	90	35.4	±	2.6
overweight	260	38.85	±	8.19	260	40.32	±	13.67	253	33.58	±	5.89	239	20.78	±	3.11	239	3226.18	±	412.45	239	36.3	±	2.2
obese	170	31.28	±	8.33	168	31.9	±	11.92	116	30.52	±	3.43	149	18.55	±	2.94	149	3924.09	±	525.28	149	36.6	±	1.7
Total	532	36.79	±	9.06	528	37.59	±	13.34	482	33.56	±	6.01	478	20.22	±	3.23	478	3345.89	±	623.74	478	36.2	±	2.2

Data a represented as means ± standard. n size vary due to metrics not completed or missing observation for the participant(s). BMI = Body Mass Index; cm = Centimeters; kg = Kiligrams; min = Minutes; mL = Mililiters; N = sample size; VO2Max = Maximal Oxygen Uptake; PAPw = Peak Anaerobic Power Output.

## Data Availability

No new data were created or analyzed in this study. Data sharing is not applicable to this article.

## Abbreviations

The following abbreviations are used in this manuscript:

20MFST	20 m Multistage Fitness Test
ANCOVA	Analysis of Covariance
BMI	Body Mass Index
CRF	Cardiorespiratory Fitness
CVD	Cardiovascular Disease
LEO	Law Enforcement Officer
PAPw	Peak Anaerobic Power Output
PAT	Physical Ability Test
SMM	Skeletal Muscle Mass
SWAT	Special Weapons and Tactics
TSAC-F	Tactical Strength and Conditioning Facilitator
US	United States
VJ	Vertical Jump
VO_2_max	Maximal Oxygen Uptake
